# Construction and identification of influenza plasmid pool imparting high yields to candidate vaccine viruses in Vero cell at low temperature

**DOI:** 10.1111/jcmm.15672

**Published:** 2020-09-09

**Authors:** Ze Liu, Xingliang Geng, Zhaohai Cui, Weidong Li, Xia Ou, Guoyang Liao

**Affiliations:** ^1^ The Fifth Department of Biological products Institute of Medical Biology Chinese Academy of Medical Science and Peking Union Medical College Kunming Yunnan Province China; ^2^ The Department of Production Administration Institute of Medical Biology Chinese Academy of Medical Science and Peking Union Medical College Kunming Yunnan Province China; ^3^ Medical Faculty Kunming University of Science and Technology Kunming Yunnan Province China

**Keywords:** high yield, influenza, plasmid pool, vaccine, Vero

## Abstract

We generated plasmid pools for the rapid preparation of candidate vaccine strains, which could grow in the Vero cells at low temperature. Firstly, we cloned in the pHW2000 plasmid each of the eight gene segments (PB2, PB1, PA, hemagglutinin [HA], neuraminidase [NA], NS, NP, M) of two master donor strains (MDS), respectively, A/Yunnan/1/2005Vca(H3N2) and B/Yunnan/2/2005Vca(By), which had Vca phenotype (cold‐adapted phenotype in Vero cells). Secondly, the similar operation was implemented with each of the HA, NA and NP segments of circulating strains with epidemic potential (parental strains). The virus rescue techniques were employed in this study, according to the homology rate of HA segments between MDS and parental strains. Then, we harvested amount of new Vca virus strains. By transmission electron microscope, it could observe new viruses' diameter and length were from 100 to 120 nm. Importantly, these reassortant viruses could get high‐yield production in Vero cells at 25℃ from the beginning to the fourth generation, which was significantly differ from their original parental viruses. Additional, these production 16 new Vca strains could maintain enough antibody binding capacity and attenuation phenotype, which consisted with their MDS. So these plasmid pools constructed by mount of different influenza A and B virus gene fragments could present desired working performance and provide convenience and realization for more Vca reassortant virus as candidate vaccine strain if needing.

## 1 INTRODUCTION

The influenza virus is an important respiratory pathogen that infects humans, including influenza A virus (IAV) and influenza B virus (IBV).^1^ They are usually predispose to exacerbation of underlying disease or development of secondary bacterial infections, especially for pregnant women, older people and young children.^2^, ^3^ Every year, there are between three and five million cases of severe illness and around 500 000 deaths worldwide.^4^, ^5^, ^6^ Additionally, the economic burden of influenza is considerable. In the United States, for instance annual costs of 0.4 billion dollars in healthcare utilization and 16.3 billion in work absenteeism are caused by influenza.^7^, ^8^, ^9^, ^10^

Vaccination is the most effective way to prevent infection and severe outcomes caused by influenza viruses.^11^, ^12^ However, serious problems with influenza vaccines are antigenic drift and antigen switching between candidate vaccine strains and seasonal strains or epidemics strains. Since 1971, World Health Organization (WHO) has provided formal recommendations for the composition of seasonal influenza vaccines.^13^ Among all vaccines, the process of making influenza vaccines is considered uniquely complicated and difficult.^14^, ^15^

Viral gene reassortment is a phenomenon of generation of new viral strains in a cell infected with more than one strain of a split genome virus. It is usable as a tool for the purposeful production of novel viral strains. Influenza viruses, due to having a split genome, are subject to this production method.^16^

Technically, two major approaches to generate new reassortant viruses, classical reassortment and reverse genetics exist at present. As for classical approach, its goal is to combine the desired hemagglutinin (HA) and neuraminidase (NA) viral segments from circulating strain with genes from master donor strain (MDS). After inoculating two influenza strains of the same type (A or B) in a 9‐day‐old embryonated chicken egg, there is a high probability of simultaneous infection of cells with both strains and intracellular reassortment of viral segments. Theoretically, there might be as many as 256 possible gene combinations. Researchers search the many combinations for the influenza strain that contains the HA and NA genes from parent strain and genes from MDS that ensure that it is able to grow efficiently in eggs or cells. Finally, this new reassortant strain will make up next year's vaccine.

If used reverse genetics approaches, scientists clone the HA and NA genes from parent strain into plasmid (eg pHW2000). Additional plasmids are created using the remaining six genes found in MDS. Then, scientists transfected the HA and NA plasmids from parent strain and the six plasmids carrying genes from MDS into animal cells growing in the laboratory.

However, at early time, it carried out influenza virus rescue based on 16 or 12 plasmids systems. It needed the infection with helper viruses of transfection of supplemental plasmids encoding polymerases. But, the efficiency of virus rescue was often not enough for vaccine industrial production. Until 2000, Hoffmann E. established the eight‐plasmid transfection system, which was with wider range of its practical. The length of the time period between the emergence of a new pathogenic strain and the preparation of a vaccine is a crucial variable in the effectiveness of a vaccination programme. The ability to generate viruses by cloning only eight plasmids reduces the time needed for the generation of potential vaccine candidates and improves existing reverse genetics systems by simplifying virus creation and reducing the overall cost of production of a vaccine.^17^ Finally, the eight gene plasmids instruct the animal cells to make the desired new influenza strain.

However, the reality is usually that most wild‐type seasonal influenza viruses encoding the recommended HA and NA antigens for immunization grow poorly in eggs even after sequential passages in the laboratory.^18^, ^19^, ^20^ In the late 1960s, Kilbourne mitigated this problem by exploiting the exceptional replication efficiency of the laboratory adapted A/Puerto Rico/8/1934 (PR8) virus in eggs through genetic reassortment method.^20^, ^21^

Most of the inactivated seasonal influenza vaccines manufactured since the early 1980s utilize PR8‐derived high‐yield viruses that include only the HA and NA segments from the seasonal circulating influenza A strains (6:2 segment ratio approach).^22^ That means six gene segment (PB1, PB2, PA, NP, NS and M) were from MDS virus strains (as PR8) and other two gene segments (HA and NA) from parental virus strains.

Recently, more research found that distinct nucleotide and amino acid sequence polymorphisms arising as a consequence of diverse passage histories may impact the antigen yields of new viruses in manufacturing.^23^

The long vaccine production process (9 months for a cycle)^24^ is largely caused by the use of chicken egg‐based vaccine production technologies, and this duration creates a window of opportunity for new virus variants to emerge, often resulting in decreased vaccine efficacy.^25^ The egg‐based vaccines prone to mutations and changes in antigenicity during egg adaptation; long production time; dependent on egg supply, which might be limited in the case of pandemics.^26^, ^27^

Regarding the reverse genetic methods had rapid production and scale‐up, production can be initiated from virus genetic sequence without live virus and no risk of mutations from laboratory passage.

In this study, we constructed a useful plasmid pool used for reverse genetic methods to prepare new influenza A and B viruses. We describe a new reassortant influenza strains production system, based on new sets of plasmids generated from circulating viruses and from MDS that are different from the egg‐adapted PR8 system. This extensive influenza practical plasmid pool would produce high‐yield candidate flu vaccine in Vero cell at low temperature. We also performed the quality control for each plasmid and determined the optimal segment ratio for each reassortant virus strain The 6:2 ratio was suitable for some high homology of HA gene, but 5:3 ratio was suitable for some particular strains, by using gene sequence comparison software (BLAST).

## 2 MATERIALS AND METHODS

### 2.1 Viruses

#### 2.1.1 Parental virus

Totally, 16 influenza virus strains were enrolled in this study initially, as parental virus. Their information of attribute and classification was listed in Table 1. In order to ensure strain diversity, the parental virus strains used in the plasmid pool construction originated from three main sources, respectively, and WHO recommends seasonal influenza in the Northern Hemisphere, avian influenza in China mainland and wild‐type human influenza, which were isolated from Yunnan Local epidemic strains. Because of the co‐circulation of geographically segregated H1N1 lineages, all seasonal H1N1 viruses were divided into preceding and posting the 2009 pandemic groups.^27^

**TABLE 1:** The information of used parental virus and reassortment rate

Virus name	Abbreviation	Virus sources	Subtype	Attribute Using	Years	Reassortment success rate
B/Brisbane/60/2008	BS/08B	WHO recommends seasonal influenza	Bv	6 + 2	2015‐2018	6/12
B/Phuket/3073/2013	PK/13B	WHO recommends seasonal influenza	By	6 + 2	2015‐2018	7/12
A/California/7/2009	CF/09H1	WHO recommends seasonal influenza	H1N1‐pdm09	5 + 3	2015‐2017	8/12
A/Michigan/45/2015	MC/15H1	WHO recommends seasonal influenza	H1N1‐pdm09	5 + 3	2017‐2018	8/12
A/Switzerland/9715293/2013	SZ/13H3	WHO recommends seasonal influenza	H3N2	6 + 2	2015‐2016	12/12
A/Hong Kong/4801/2014	HK/14H3	WHO recommends seasonal influenza	H3N2	6 + 2	2016‐2018	12/12
A/Solomon Islands/3/2006	SL/06H1	WHO recommends seasonal influenza	H1N1 pre‐pdm09	6 + 2	2007‐2008	10/12
A/New Caledonian/20/99	NC/99H1	WHO recommends seasonal influenza	H1N1 pre‐pdm09	6 + 2	2005‐2006	10/12
A/Yunnan/11/2015(H5N1)	YN/15H5	avian influenza in China mainland	H5N1	6 + 2	2015	6/12
A/Guangdong/61/2016(H9N2)	GD/16H9	avian influenza in China mainland	H9N2	5 + 3	2016	6/12
A/Shanghai/1/2016(H7N9)	SH/16H7	avian influenza in China mainland	H7N9	5 + 3	2016	6/12
A/Anhui/5/2017(H7N9)	AH/17H7	avian influenza in China mainland	H7N9	6 + 2	2017	5/12
B/Hongta/22/2008(By)	HT/08B	wild‐type human influenza in Yunnan	By	6 + 2	2008	7/12
B/Longyang/17/2015(Bv)	LY/15B	wild‐type human influenza in Yunnan	Bv	6 + 2	2015	7/12
A/Kunming/11/2017	KM/17H3	wild‐type human influenza in Yunnan	H3N2	6 + 2	2017	12/12
A/Chuxiong/8/2017	CX/17H1	wild‐type human influenza in Yunnan	H1N1‐pdm09	5 + 3	2017	6/12

#### 2.1.2 Master Donor strains (MDV)

A/Yunnan/1/2005Vca(H3N2) (abbreviated: *YN/05H3Vca*)^28^ and B/Yunnan/2/2005Vca(By)^29^, ^30^ (abbreviated: *YN/05Bvca*). Both of them were isolated from patients' samples in the Yunnan Province of China in 2005. They have been extensively studied during the last decade. They have the double phenotype of cold adaption (ca) and Vero cell adaption (va), and it was stored at the Institute of Medical Biology, Chinese Academy of Medical Sciences. When passaged them to the 110th generation, it could maintain high yield in Vero cells at 25°C. Additionally, its lower virulence was assessed by animal experiments, which were certified.^28^, ^29^

### 2.2 Construction of the gene plasmid pool

Total RNA was extracted from influenza virus culture solution (parental strain in Madin‐Darby canine kidney (MDCK); MDS in Vero) using the QiAmp Viral RNA minikit (Qiagen, Valencia, CA) and reverse transcribed to cDNA and amplified using a one‐step reaction system, and it was one‐step RT‐PCR kit, which was purchased from Qiagen (cat. no. 6180).

These gene segments have been obtained through RT‐PCR method. Each pair of primers for each fragment is listed in Table 2. Then, all gene fragments were sequenced by Shanghai Biological Engineering Technology Services Ltd (Shanghai). We analysed the HA, NA and NP genes by using gene sequence comparison software (BLAST). The genetic distance and phylogeny analysis were calculated. Here, we employed the MEGA software online, which is molecular evolutionary genetics analysis software^31^ and online gene sequence comparison software, called BLAST^32^ for our research.

**TABLE 2:** Generic pairs of primers used for the PCR amplification of viral segments of the type A and B parental and master donor influenza strains used in the study

Primer name	Sequence(5ʹ‐3ʹ)	Size (bp)	Primer name	Sequence (5ʹ‐3ʹ)	Size (bp)
A‐PB2‐F	ACCTCCGAAGTTGGGGGGGAGCAAAAGCAGGTCAATTAT	39	B‐PB2‐F	CCGAAGTTGGGGGGGAGCAGAAGCGGAGCGTTTTCAAG	40
A‐PB2‐R	TTGGGCCGCCGGGTTATTAGTAGAAACAAGGTCGTTT	37	B‐PB2‐R	TTGGGCCGCCGGGTTATTAGTAGAAACACGAGCATT	33
A‐PB1‐F	ACCTCCGAAGTTGGGGGGGAGCAAAAGCAGGCA	33	B‐PB1‐F	CCGAAGTTGGGGGGGAGCAGAAGCGGAGCCTTTAAGATG	38
A‐PB1‐R	TTGGGCCGCCGGGTTATTAGTAGAAACAAGGCATTT	36	B‐PB1‐R	TTGGGCCGCCGGGTTATTAGTAGAAACACGAGCCTT	33
A‐PA‐F	ACCTCCGAAGTTGGGGGGGAGCAAAAGCAGGTAC	34	B‐PA‐F	ACCTCCGAAGTTGGGGGGGAGCAGAAGCGGTGCGTTTGA	34
A‐PA‐R	TTGGGCCGCCGGGTTATTAGTAGAAACAAGGTACTT	36	B‐PA‐R	TTGGGCCGCCGGGTTATTAGTAGAAACACGTGCATT	33
A‐HA‐F	ACCTCCGAAGTTGGGGGGGAGCAAAAGCAGGGG	33	B‐NP‐F	CGAAGTTGGGGGGGAGCAGAAGCACAGCATTTTCTTGT	38
A‐HA‐R	TTGGGCCGCCGGGTTATTAGTAGAAACAAGGGTGTTTT	36	B‐NP‐R	GGGCCGCCGGGTTATTAGTAGAAACAACAGCATTTTT	36
A‐NA‐F	ACCTCCGAAGTTGGGGGGGAGCAAAAGCAGGAGT	29	B‐M‐F	ACCTCCGAAGTTGGGGGGGAGCAGAAGCACGCACTT	41
A‐NA‐R	TTGGGCCGCCGGGTTATTAGTAGAAACAAGGAGTTTTTT	36	B‐M‐R	TTGGGCCGCCGGGTTATTAGTAGAAACAACGCACTT	40
A‐NP‐F	ACCTCCGAAGTTGGGGGGGAGCAAAAGCAGGGTA	34	B‐NS‐F	CGAAGTTGGGGGGGAGCAGAAGCAGAGGATTTGTTTAGT	40
A‐NP‐R	TTGGGCCGCCGGGTTATTAGTAGAAACAAGGGTATTTTT	39	B‐NS‐R	GGGCCGCCGGGTTATTAGTAGTAACAAGAGGATTTTTAT	38
A‐M‐F	ACCTCCGAAGTTGGGGGGGAGCAAAAGCAGGTAG	34	B‐HA‐F	ACCTCCGAAGTTGGGGGGGAGCAGAAGCAGAGCA	29
A‐M‐R	TTGGGCCGCCGGGTTATTAGTAGAAACAAGGTAGTTTTT	39	B‐HA‐R	GGGCCGCCGGGTTATTAGTAGTAACAAGAGCATTTT	35
A‐NS‐F	ACCTCCGAAGTTGGGGGGGAGCAAAAGCAGGGTG	34	B‐NA‐F	ACCTCCGAAGTTGGGGGGGAGCAGAAGCAGAGCA	29
A‐NS‐R	TTGGGCCGCCGGGTTATTAGTAGAAACAAGGGTGTTTT	38	B‐NA‐R	GGGCCGCCGGGTTATTAGTAGTAACAAGAGCATTTT	35

The eight gene fragments of the two donor strains were inserted into the bidirectional expression plasmid *pHW2000*,^17^ whose schematic with markings is shown in Figure S1. The three gene fragments (HA, NA, NP) of the parental strains were also inserted into *pHW2000* by the same methods. The homologous recombination method was in accordance with our previous research^33^ and that of Ljungberg et al.^34^ After harvesting each gene fragment, we incubated them with the linearized plasmid at 37°C for 30 minutes in the presence of a homologous recombinase (using the ClonExpress Entry One Step Cloning Kit, Cat. No. C114‐01/02), which was purchased from the Vazyme Biotechnology Co., Ltd. (Nanjing, China), then transformed into competent *Escherichia coli* and screened for positive clones. The DH5α strain of *E coli* was used, with ampicillin selection marker. The identity of plasmids extracted from positive bacterial clones was confirmed by PCR, digestion by restriction enzymes and sequencing.

### 2.3 Quality control of plasmid pool

After construction of plasmid pool, we performed quality control for all involved plasmid. Inclusion criteria were as follows: (a) single dose volume ≥ 500 μL; (b) concentrations range between 200 and 500 ng/μL; (c) ratio of A260/A280 between 1.9 and 2; and (d) ratio of A260/A230 between 2.3 and 2.9.

Exclusion criteria were as follows: the PCR amplification of the plasmid encoded viral gene either (a) did not generate a full‐length product; or (b) generated more than one product.

### 2.4 Suitable ratio selection

For the production of reassortant Vca strains, we decided the use of a 6:2 or 5:3 ratio between parental and MDS segments based on the degree of sequence identity of HA genes when compared using gene sequence comparison software (BLAST). By pre‐experimental founding, we set a useful bar of the identities value as 97%. Compared their whole HA gene, it could use 6:2 ratio to reassort new virus when identity value was above 97%. If it was under 97%, it chose 5:3 ratio to generate the new target virus, regardless of the source of parental strains. Comparing with the virus pool strains, calculated the genetic distance and phylogenetic analysis, their phylogenetic distance should be under 10%, whenever using 6:2 or 5:3 ratio. It needs to be mentioned that the comparison of HA gene sequences was performed over the whole HA gene, and not on just a part of it.

### 2.5 HEK 293T transfection and propagation on embryonated chicken egg

To select the appropriate sets of 8 gene fragments and transfected them into HEK 293T cell, the given steps were as follows: (a) plate 1 × 10^5^ cells per well in 0.5 mL of complete growth medium in 24‐well plate. It employed the DMEM/F12 (Gibco，New York) as a complete growth medium, which contained 10.00% calf serum, 100 U/mL penicillin, 100 mg/mL streptomycin, 2 mmol/L L‐glutamine and 25 mmol/L HEPES. Cell density should be 80% confluent on the next day for transfection; (b) for each well of cells to be transfected, dilute 0.5 μg of DNA in 100 μL of Opti‐MEM Reduced Serum Media without serum and added 1 μL of Lipofectamine LTX Reagent into it^35^ (here, the Lipofectamine™ LTX Reagent with PLUS™ Reagent (cat. no. 15338100) was purchased from the Thermo Fisher Scientific; The Opti‐MEM^®^ I Reduced Serum Medium (cat. no. 31985070) was purchased from Gibco, China); (c) then mixed gently and incubated 30 minutes at room temperature to allow for the formation of DNA‐Lipofectamine complexes and after 30‐minute incubation, add 100 μL of this complexes directly to each well containing cells and mix gently by rocking the plate back and forth; and (d) incubated the cells at 37°C in a 5% CO_2_ incubator for 48 hours post‐transfection, then harvested all medium.

From each plate, 12 mL of virus suspension could be harvested and then injected it into 12 eggs by the dose of 1mL per egg. Viruses were grown in 9‐day‐old embryonated chicken eggs at 33°C for 72 hours. Allantoic fluid was harvested, clarified by centrifugation at 4696 g for 30 minutes and stored at −70°C. The reassortment success rate was listed in Table 1.

### 2.6 Serial passages in Vero cells at 25°C

We seeded the 0.1 mL of new reassortment virus with 10^7^ p.f.u./mL on Vero cells in the *T*_25_ flask. When 80% cytopathic effect (CPE) was observed, we harvested all of the virus culture medium with 10 mL. Then, we detected its viral titter by plaque assay in Vero cells and seeded it for the next passage. The conditions for the Vero cells culture were according to our previous publication.^30^

As a parallel test, we cultured these original parental virus strains at the same condition. Simultaneously, it should note that 0.4 μg/mL L‐1‐Tosylamide‐2‐phenylethyl chloromethyl ketone (TPCK)‐trypsin was added once every 72 hours to help cleave its HA protein.^36^ And it has confirmed that adding TPCK‐trypsin at this concentration does not cause CPE at this time point. After 96 hours of culture, we harvested all culture solution to test the influenza virus HA titre, to further confirm no enough virus replication here by HA test.

### 2.7 Mass production of reassortant viruses

The mass production of reassortant virus in Vero cells and parental virus in MDCK cells was implemented by cell factory (it had 6000 cm^2^, which were purchase from NUNC, NALGENE, Thermo Scientific Finnpipette, cat no. 170009，Rochester，USA). The live virus was purified by ultrafiltration. And the condition of cell factory culture influenza virus and ultrafiltration purification process were consisted with Xu's research^37^ in our department. In briefly, virus harvest fluid is clarified using 0.65‐, 0.45‐ and 0.22‐μm filter cartridges, concentrated with 300 KD UF membrane; benzonase treatment at 37°C for 4 hours and concentrated with 100 KD Ultra‐Filtration (UF)membrane below 5psi and chromatographic purification with Sepharose 4 fast flow (FF) and diethylaminoethyl (DEAE) Sepharose FF. Finally, Vero cell‐derived influenza vaccine reaches Chinese pharmacopoeia requirements.

### 2.8 Single radial immunodiffusion

#### 2.8.1 Serum source

Firstly, we got four subtypes standard antiserum from Chinese National Influenza Center, as H1 (goat anti A/beijing/341/2018), H3 (goat anti A/sichuan/514/2018), BV (goat anti B/chongqing/118/2018) and BY (goat anti B/tianjing/442/2018). And these types of serum had been treated and prepared by Diagnostic department of Chinese National Influenza Center.

Secondly, we collected some influenza patients' serum at their recovery period (3 weeks post‐infection) in Yunnan Province Hospital. The pathogens of these patients after influenza infection were B/Hongta/22/2008(By), B/Longyang/17/2015(Bv), A/Kunming/11/2017 and A/Chuxiong/8/2017, respectively.

#### 2.8.2 Serum preparation

We collected 2‐3mL fasting venous blood, then separated the serum by centrifugation with 2301 g for 20 minutes and stored the patient sera at −20°C for further use. These sera were treated with receptor destroying enzyme (Denka Seiken, Tokyo, Japan) to remove non‐specific agglutination inhibitors, and it was heated to 56°C for 1 hour to inactivate its complement.

For the procedure of immunodiffusion testing, here, we standardized the human antibodies to 1:320, according to HA inhibition testing by that infection virus strain, then mix this antibody with 1% agarose gel in 0.01 mol/L phosphate belanced solution (PBS) at 50°C and prepare a gel plate. Different human antibodies matched with different virus subtypes had been prepared as different gel plates, and placed each virus strain on each well in that plate. For example, the H3 type virus should to be added the well of 1% agarose gel plate contain H3 type serum, but not H1 or B type.

#### 2.8.3 Sample preparation

We diluted each virus strain by 0.01 mol/L PBS to 32 HA units, then treated them with Zwittergent 1% (w/v) (Merck, Poseung‐Eup,South Korea.) for 30 minutes at room temperature.

##### Immunodiffusion

Agar gel immunodiffusion (AGID) testing was used as an immunological technique for the detection and identification of different viral antigens. A 1% agarose gel plate contains special serum that was cut to form a series of wells in the gel.

Each 50 μL virus strain sample was placed in one well, according to different type or subtypes. Herein, the thickness of agarose gel was 4 mm; the diameter of the wells in the agarose gel was 5 mm. Then, plates were incubated at 37°C for 24 hours. During this time, the antigens could diffuse around the well and could be recognized by antibodies. Then at a weight of 25 pounds, the 1% agarose gel plate was pressed to a 0.5‐mm‐thick sheet and the excess water was removed between eight layers of clean filter paper.

##### Staining

It was a visual signature of antigen recognition. Staining solution (0.4% Coomassie Brilliant Blue R250) was carried out for 25 minutes at 37°C, which was purchased from Solarbio (cat. no. C8430, Beijing, China); decolorizing solution (absolute ethanol: glacial acetic acid: water = 29:12:59 by volumetric ratio) was carried out for 20 minutes at 37°C.^38^, ^39^

##### Measurement

After incubation, the immunodiffusion ring can be stained with coomassie brilliant blue. At last, vernier calliper used to measure these circle diameter of each viruses' immunoprecipitated circles and to compare their differences.

### 2.9 Attenuation phenotype of reassortant virus

There were three main test parameters to be calculated: median lethal dose (LD_50_), minimal lethal dose (LD_01_) and maximal tolerance dose (LD_0_), respectively. The minimal lethal dose (LD_01_) was the least amount of virus that can produce death in Balb/c mouse under controlled conditions; the maximal tolerance dose (LD_0_) was the highest dose of attenuated virus that could produce the desired effect without unacceptable toxicity or death.

A total of 240 female BALB/c mice at 8 weeks old were randomly used to taken a testing. Each mice received nasal inoculation with a different virus strain after anaesthesia by sodium pentobarbital (60‐80 mg/kg). LD_50_, a dose required to kill 50% of mice, was calculated following the previously described methods for determination.^40^

This study was performed in strict accordance with the recommendations in the Guide for the Care and Use of Laboratory Animals of the National Institutes of Health. The protocol was approved by the Committee on the Ethics of Animal Experiments of the Peking Union Medical College (permit number: PUMC201623‐097663). All of the surgeries were performed under sodium pentobarbital anaesthesia, and all efforts were undertaken to minimize suffering.

Under the supervision of the animal ethics committee of Peking Union Medical College, if animal lost more than 20% of their initial weight during our research after nasal inoculation, they were killed with humanitarian methods.

### 2.10 Statistical analysis of experimental data

All of the data are represented as the mean ± SD of three or more independent experiments. Since the data were homogenous, analysis of variance, the Student‐Newman‐Keuls test and Pearson's correlation were used. All of the analyses were performed using SPSS software, version 20.0 (SPSS Inc., Chicago, IL, USA). *P* values less than 0.05 were considered to be statistically significant, and less than 0.01 were considered to be very significant.

## 3 RESULTS

### 3.1 Identification of the target gene fragments

**FIGURE 1:**
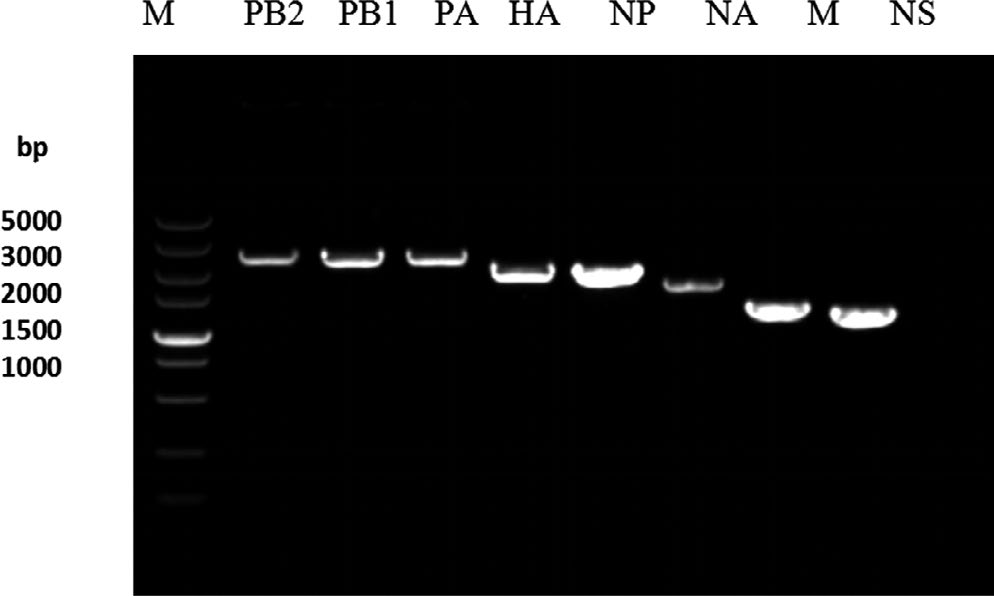
The results of 1% agarose gel electrophoresis with eight target typical gene plasmids by PCR detecting for these plasmid pools

We generated 64 unique plasmids: 48 plasmids encoding each of the HA, NA and NP segments of the 16 parental virus strains and 16 plasmids encoding each of the eight segments of the two MDS viruses. Their ID was given information of species resources, gene name and location of conservation sites. They were used in the reassortant virus production. The Figure 1 showed the results of agarose gel electrophoresis of PCR products amplified from a set of eight plasmids produced in this way. It could observed these plasmids could had each single clear electrophoretic bands with correct size.

### 3.2 Observation of virus particles by transmission electron microscope

**FIGURE 2:**
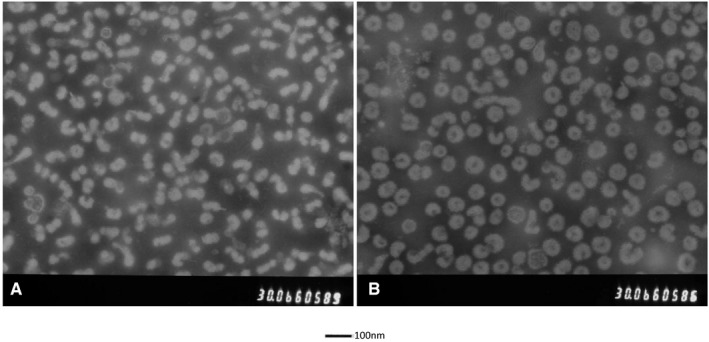
Observation of virus particles by transmission electron microscope. The morphology was complete, and the background was clear. The envelope structure of the virus was clearly visible and showed typical spherical shape influenza virus particles. A, The new reassortant strain; (B) the parental strain

Using 2% phosphotungstic acid as the washing liquid for background, the reassortant viruses were observed using an electron microscope. The procedure of preparation of virus samples for EM and the procedure were described in our previous research.^41^, ^42^, ^43^ The diameter and length of these viruses were approximately from 100 to 120 nm (Figure 2A). The morphology was complete, and the background was clear. The envelope structure of the virus was clearly visible and showed typical spherical shape influenza virus particles. Its size and shape were similar to that of the parental strain (Figure 2B), indicating that reverse genetic to reassortant influenza virus had a normal and complete morphological structure.

### 3.3 Growth characteristics of 16 reassortant viruses in Vero cell at 25°C

**FIGURE 3:**
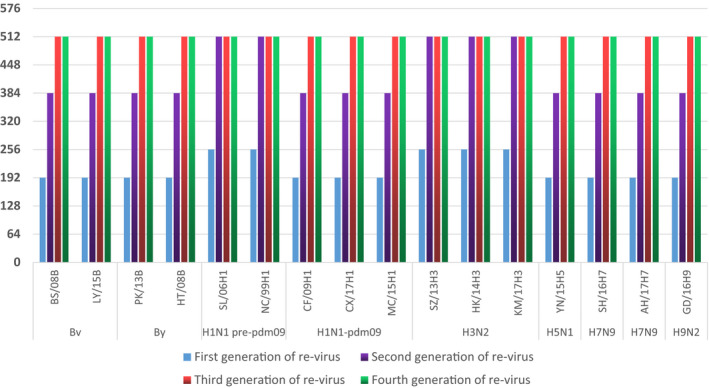
The virus yield change trend of 16 reassortment virus in Vero cell at 25°C by different types

We cultured these 16 reassortant virus and the original parental virus in Vero cells at 25°C, respectively, from the first to fourth passages in a parallel testing. Then, we calculated their viral titter in Vero cells, illustrating these changes trend in Figure 3. These reassortant viruses could get high‐yield production in Vero cells at 25°C from the beginning to the fourth generation. However, the original parental viruses could not be serially passaged in Vero cells at 25°C, which declined from the second generation. As shown, the reassortant virus showed a 100‐fold increase in growth titters by the fourth serial passage. This results could be in facilitating industrial production.

### 3.4 AGID test

**FIGURE 4:**
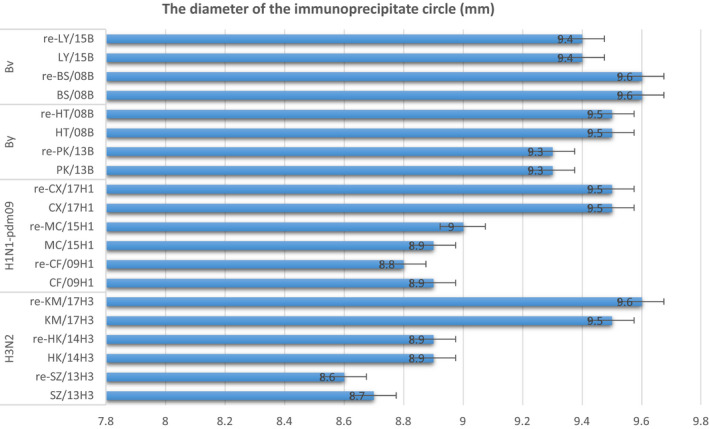
The results of agar gel immunodiffusion test. The distribution of diameter for each virus matched human antibodies. All data were represented as means ± SD ($\bar{x}$ ± *s*) of three or more independent experiments

Figure 4 illustrated that the diffuse of specially infected virus and its matched antibodies were always above 9.4 mm, by diameter. Antigen phenotype determines the size of the diameter of the immunoprecipitated circle, the higher the matching, the greater the diameter (figure about single radial immunodiffusion as Figure S2, which showed the H3N2 subtype as a example). In the scenario with faster antigenic drift, differences in the value of the circle diameter were obvious (H3N2). But the B type virus with lower antigenic drift had little change of diameter between strains. More importantly, it found that there was no significant difference in the value of circle diameter between original virus and reassortant virus. It means that our plasmid pool could provide feasibility for new *Vca* reassortant virus, maintained its antigen phenotype.

### 3.5 Identification of attenuation of reassortant virus in mice

The tissue culture infected dose 50% (TCID_50_) of each virus strain was detected according to the methods reported by Reed and Muench in 1938^26^; here, we employed the Vero cell for testing. All experiment virus were evaluated by Balb/c mouse.

We obtained the reassortant viruses by using reverse genetics methods. The values of LD_50_ were 1000‐fold lower in parental strains than in reassortant strains. It illustrated these two donor virus strains had provided a safety backbone for future using. As for LD_01_ and LD_0_, it also found this trend. The minimal lethal dose (LD_01_) could increase from 10^3^ to 10^4^, and the maximal tolerance dose (LD_0_) increased from 10^4^ to 10^7^ after reassortment experiment (Table 3).

**TABLE 3:** The results of identification of attenuation of reassortment virus, present by median lethal dose (LD50), minimal lethal dose (LD01) and maximal tolerance dose (LD0)

Virus type	Virus name	LD50: median lethal dose		LD01: minimal lethal dose		LD0: maximal tolerance dose	
		original strain	reassortment strains	original strain	reassortment strains	original strain	reassortment strains
influenza B virus	BS/08B	7.33E + 03	8.01E + 06	3.14E + 03	6.89E + 04	2.45E + 04	3.59E + 07
	LY/15B	7.36E + 03	7.88E + 06	3.44E + 03	6.90E + 04	2.41E + 04	3.62E + 07
	PK/13B	7.40E + 03	7.80E + 06	3.37E + 03	6.88E + 04	2.38E + 04	3.68E + 07
	HT/08B	7.38E + 03	7.91E + 06	3.38E + 03	6.88E + 04	2.42E + 04	3.61E + 07
		*t* = 128.9		*t* = 610.4		*t* = 137.1	
		*P* < 0.0001		*P* < 0.0001		*P* < 0.0001	
Influenza A virus	SL/06H1	6.48E + 03	7.07E + 06	1.99E + 03	6.11E + 04	1.45E + 04	2.02E + 07
	NC/99H1	6.56E + 03	7.27E + 06	1.91E + 03	6.12E + 04	1.39E + 04	2.09E + 07
	CF/09H1	6.12E + 03	7.33E + 06	1.83E + 03	6.19E + 04	1.87E + 04	2.13E + 07
	MC/15H1	6.43E + 03	7.00E + 06	1.87E + 03	6.09E + 04	1.84E + 04	2.09E + 07
	CX/17H1	6.82E + 03	7.31E + 06	1.81E + 03	6.11E + 05	1.61E + 04	2.08E + 07
	SZ/13H3	7.12E + 03	7.57E + 06	2.10E + 03	6.31E + 04	1.64E + 04	2.20E + 07
	HK/14H3	7.17E + 03	7.64E + 06	2.30E + 03	6.42E + 04	1.49E + 04	2.12E + 07
	KM/17H3	7.18E + 03	7.33E + 06	2.33E + 03	6.44E + 04	1.44E + 04	2.07E + 07
	YN/15H5	6.09E + 03	6.48E + 06	1.00E + 03	6.09E + 04	1.13E + 04	1.37E + 07
	SH/16H7	6.32E + 03	6.44E + 06	1.54E + 03	6.19E + 04	1.68E + 04	1.61E + 07
	AH/17H7	6.40E + 03	6.29E + 06	1.56E + 03	6.20E + 04	1.65E + 04	1.59E + 07
	GD/16H9	6.11E + 03	6.52E + 06	1.61E + 03	6.12E + 04	1.78E + 04	1.39E + 07
		*t* = 51.60		*t* = 2.317		*t* = 21.15	
		*P* < 0.0001		*P* = 0.0302		*P* < 0.0001	

Additionally, the weight loss is a critical indicator in vaccine safety evaluation. Recorded the weight of all experiment animals every day. And the results were consistent with PhD Long's research.^18^ All parental strains could directly conduct weight loss exceeded 20%. But after reassortment method production, if infected with the new strains, the animals were in good condition. Their weights grew slowly and steady, illustrating that reassortant virus was safe for live attenuated influenza vaccines (LAIVs) development.

## 4 DISCUSSION

### 4.1 Vaccination

Influenza viruses cause annual seasonal epidemics and occasional pandemics of human respiratory disease, which represent a serious public health and economic problem. Fortunately, it could be most effectively prevented through vaccination.^13^ But there is a challenge here, that influenza viruses suffer continual antigenic variation, which requires either the annual reformulation of seasonal influenza vaccines or the rapid generation of vaccines against potential pandemic virus strains.

There are two methods to process influenza vaccine virus selection and development, reverse genetics and classical reassortment approach. Their goal is to combine the desired HA and NA genes from circulating strain with the six other genes from MDS, which grows well in eggs or cell cultures.

### 4.2 New MDS

The widely used master donor virus (MDV) is the A/Puerto Rico/8/1934 (PR8) because of its high growth reassortant (HGR) characteristics in eggs.^44^ Other MDV strains are also currently developed for LAIV vaccines: A/Ann Arbor/6/60 (H2N2),^45^ B/Ann Arbor/1/66^46^ and A/Leningrad/134/17/57 (H2N2) ^47^ influenza strain. In this study, we employed YN/05H3Vca and YN/05Bvca as MDV for construction of plasmid pool because of their double phenotype of ca and va. And their attenuated character was also confirmed.^48^, ^49^

### 4.3 Vero cells adaptation

First of all, the suitable cell line is very important to study the virus adaptability. We employed the Vero cell line in this study. But it is true that the advantages of the use of the interferon‐deficient Vero cell line for the production of candidate vaccine strains, which is in order to decrease the fitness of the virus to interferon‐competent mammalian cell. Vero E6 might be more useful to research adaptation influenza virus. Vero E6 cells show some contact inhibition, so these are suitable for propagating viruses that replicate slowly. In fact, Vero or Vero E6 cells do not secrete interferon‐alpha or interferon‐beta when infected by viruses.^50^ But if interferon from another source is added to the culture, the process of virus‐infected Vero E6 cells will be interfered, because they have the interferon‐alpha/beta receptor. The defect is due to a ~9‐Mb deletion in chromosome 12 of Vero cells, causing the loss of the type I interferon gene cluster.^51^ Actually, the Vero E6 could be a useful cell infection influenza model in some field. But if considering vaccine production and its application, the Vero E6 cells are not approved as vaccine production cell line in China's vaccine production regulations. Admittedly, this is still a very valuable attempt for our department in the future.

Up to now, indeed, a great amount of results was reported for influenza production in Vero cells at 37℃ and even at 25℃. From University of Natural Resources and Applied Life Sciences' research, after 20 passages, the wild‐type strain A/Singapore/1/57 (H2N2) could reached the titre 2.0 × 10^8^ pfu/mL in Vero cell line, which had been defined as Vero cell adaption. Subsequently, it was adapted to the growth at a suboptimal temperature 25°C, following 25 passages. At last, it was defined as Vero and cold adaption virus, Vca, which reached the titre of 7.0 × 10^8^ pfu/mL at 25°C.^52^ By our previous study, our MDV reached the titre of 8.4 ± 0.12 Log 10 TCID_50_ at 33°C, but 7.1 ± 0.08 Log 10 TCID_50_ at 25°C.^18^ There was difference between A/Yunnan/1/2005Vca(H3N2) and A/Singapore/1/57 (H2N2) in virus titre distribution trend when used as Vca. Additionally, there is no dextran in the virus culture solution. When passaged A/Yunnan/1/2005Vca(H3N2) to the 110th or more generation, it could maintain high yield in Vero cells at 25℃. If opportunity, we are very willing to discuss and research with the PhD Julia Romanova in the future.

During our another research project, we explore the reasons of high replication in Vero cells at 25℃, which is undergoing now. And we are very pleasure to learn from the famous scientists. In PhD O'Callaghan RJ's research,^53^ it showed that infected Vero cells produced non‐infective influenza that remained predominantly cell associated. But the infectious virus particles could be prepared through HA titre testing and TCID_50_ testing in our research. Honestly, we study influenza A and B virus strain, but not C type, which was not used as vaccine. It was a limitation of our research.

PhD Kaverin NV^54^ found that influenza virus replication in Vero cells was impaired by rapid inactivation of trypsin in the culture fluid by a factor secreted by Vero cells and that repeated addition of trypsin to the culture medium allows for multicycle growth of influenza A. In fact, not only influenza A but also B type virus needed trypsin during their replication in Vero cells.

In the same year, PhD Govorkova EA^55^ suggested that by adding trypsin the efficiency of primary isolation for circulating H3N2 strains was similar in Vero and MDCK cells and that the amino acid sequence of HA1 was similar if passaging in Vero cells or MDCK. This is a great try and founding. Our goal is in high‐yield industrial production, including but not limited to isolation of influenza virus.

### 4.4 Reverse genetics method using

As for the reverse genetics method, we employed the *pHW2000* cloning system, which was carried out in the laboratory to generate reassortant viruses as vaccine candidate strains. This cloning system has the advantage of including promoters for pol I and pol II in opposite directions on the sides of the cloned viral segments in order to generate both mRNAs and copies of the viral genome.^56^ The target viral segment is inserted between the sites for a pol II promoter element (truncated CMV promoter) and a pol I promoter element (truncated human pol I promoter) that are present in positive and negative orientation, respectively. Therefore, from this plasmid, both viral mRNA (pol II) and vRNA (pol I) can be synthesized without the need of additional helper viruses or further transfection with other polymerase genes.

In this study, we used homologous recombination between PCR products and the *pHW2000* plasmid instead of digestion with BsmBI or BsaI and insertion into BsmBI sites in the linearized pHW2000.^17^ This method was useful during our research. Previously, Professor Gao^33^ compared the restriction enzyme digestion and homologous recombination methods to construct the pUC57 vector, and proposed that the double enzyme digestion vector construction method is cumbersome, while the homologous recombination method is relatively simple, and the vector can be constructed efficiently and quickly.

The establishment of high‐quality plasmids pool facilitated the using of reverse genetics. The DNA reverse transcribed from the RNA of the original viruses was stored in a population of similar plasmids, each containing a different insert of DNA. Using a host cell (eg *E coli*) to carry the plasmid allows for easy amplification and retrieval of specific clones from the pool for analysis. It also provided a convenient transport to different laboratory. Of course, the quality control of the plasmid pool was very important by our research founding, which described previously.^34^

In general for the production of reassortant viruses of the A/H1N1 type derived from strains circulating after 1977, the ratio between the gene segments of the MDSs and circulating strains was 6:2.^57^ Isakova‐Sivak et al have recently shown modification of the genome of reassortant vaccine viruses by incorporating the NP gene from the parental wild‐type viruses represents a simple strategy to improve influenza vaccines.^58^ It would therefore also be important to reconsider the genome composition of reassortant viruses for inactivated influenza vaccines (IIVs) and LAIV.^59^ These viruses are usually prepared using highly egg‐adapted virus PR/8, which dates back to the 1930s, and the CTL epitopes of recent viruses have also significantly evolved since then. The MDVs used in this study were back to the 2005. And these scientists strongly recommend incorporating wild‐type NP into the genome of seasonal LAIV and IIV reassortant viruses to improve cell‐mediated immune responses. The evolution of seasonal influenza viruses might be a decision‐maker in this study.^13^ If the consistent homology rate of HA was above 97%, 6:2 ratio should be taken; if it is not, 5:3 ratio should be taken.

However, on the other side, some scientists pointed out the interaction between HA, NA and internal viral proteins as being important for improved viral fitness. And it is an unignored fact these eight gene fragments should be considered as whole viruses with their genes each other interaction, especially, PB1, PB2 and PA.^44^ In our study, the reassortment success rate cannot be always 100%. We cannot rule out the presence of interaction between HA, NA and internal viral proteins. But our goal is to package viruses containing specific known and wanted genes with special growth characteristics (eg in Vero cell at 25°C). Simultaneously, our research required that the protective antigens (HA and NA) of new reassortant virus have consistency with their parent strain.

Beside, the results of previous studies we also feedback 5:3 could also play the reassortment function role by our team. When we employed the classical method, we indeed found this ratio, such as 5:3.^18^ Our goals are to generate new reassortant virus, which maintain the immunogenicity and antigenicity of the parental virus strain and inherit the growth characteristics (va and ca) of MDSs in the shorter possible time. Either 6:2 or 5:3 ratio was help us to address it.

### 4.5 High recombination efficiency

At the first generation of reassortant virus, it employed the egg to proliferate but not MDCK or Vero cells, because the Specified Pathogen Fre (SPF) chicken embryo allantoic fluid provided condition, which was more conducive to the proliferation of live virus. However, these process had not work effectively when it used 6:2 ratio under HA identities ＜97%, in our finding. Regarding one thing is clear on the whole, it needs the haemagglutinin (HA) and NA genes encoding the surface glycoproteins from their parental virus strains. Now, we recognize that NP was also important.

And according to the different gene evolution, it chose suitable formation or called gene constellation that could help us improve the rescue success rate (≥50%) in a short time.

Actually, some scientists define high efficiency from their long‐term work experience. If it is low efficiency, it might be 1 or 2 positives in 12 chicken embryos. When the influenza pandemic happen, for vaccine developers and producers, if the threshold of high recombination efficiency is raised from 10% to 50%, it will help the timely use of effective vaccines in the short term, which is conducive to the risk management of vaccine development failure. But in fact, the 50% threshold is based our views and experience. And we welcome vaccine manufacturers to discuss and develop final threshold. This is an opening definition.

This study with plasmid pool, influenza reverse genetics approaches and their implementation had supplied rapid, convenient method to rescue safe and efficacy candidate vaccine strains.

### 4.6 Further using

Theoretically, the findings of this study will not only be used for the development of LAIV, but also for the development of IIVs, particularly during seasons with vaccine shortages,^60^, ^61^ because there was significant difference in median lethal dose (LD_50_), minimal lethal dose (LD_01_) and maximal tolerance dose (LD_0_) between before and after reassortment experiment. The identification testing of attenuation of reassortant virus had positive results.

It could solve the keynote of LAIV developing with Vero cell cold‐adapted strain (Vca). These properties were optimal for efficient growth and vaccine production. Additionally, we were not only studied the IAVs but also IBVs for the plasmid pool construction to generate the reassortant viruses.

The cold adaptation characteristic of LAIV is very useful and important. The human nasal or upper respiratory tract temperature is between 25°C and 28°C, which is suitable for LAIV replication. Then, the inoculation pathway could occur through nasal immunization, which provides a convenient alternative for subjects and a similar route to natural infection. This injection route can elicit mucosal cellular immunity in addition to humoral immunity, thus providing strong protective efficacy and long‐lasting immunity.

Collectively, our study provides feasibility of rapid, quantities and high recombination efficiency for future IIV or LAIV vaccine by these plasmid pool.

### 4.7 Limitations

However, more immunization experiment and challenge testing should be taken in the future for practical protective effect against seasonal influenza or avian Influenza. Back to the origin MDV, they were differ from PR8, and they and their reassortant virus require more human clinical trails to confirm their safety and immunogenicity.

On the next step, we should analyse more data after many more passages on the Vero cell line at 25°C, which included the evolution of the viral titter; the conservation of surface antigenicity and of HA sequence; and the conservation of attenuation in vivo.

The immunogenicity of the viral strains (H5N1, H7N9, H9N2 or H1N1‐pdm09) had not been tested in immunodiffusion, that is a big stumbling stone during our research. We cannot collect the positive serum from patients infected with H5N1, H7N9 and H9N2. We have plan injecting the inactivated reassortant avian virus into the goat, to harvest the positive serum. Then, we could do AGID testing for avian virus. Maybe in the next flu season, we hope to carry out research on this project.

## CONFLICT OF INTEREST

The authors confirm that there are no conflicts of interest.

## AUTHOR CONTRIBUTION

**Ze Liu:** Data curation (lead); Investigation (equal); Methodology (equal); Writing‐original draft (lead); Writing‐review & editing (equal). **Xingliang Geng:** Formal analysis (equal); Investigation (equal); Methodology (equal); Resources (equal); Writing‐original draft (equal). **Zhaohai Cui:** Investigation (supporting); Methodology (supporting); Validation (supporting). **Weidong Li:** Conceptualization (equal); Data curation (equal); Project administration (equal); Supervision (equal). **Xia Ou:** Formal analysis (equal); Investigation (equal); Methodology (lead); Writing‐original draft (supporting); Writing‐review & editing (lead). **Guoyang Liao:** Funding acquisition (lead); Project administration (lead); Supervision (lead).

## Supporting information

Fig S1‐S2

## Data Availability

All data, models and code generated or used during the study appear in the submitted article.
