# Supplementary material for: Construction and identification of influenza plasmid pool imparting high yields to candidate vaccine viruses in Vero cell at low temperature
Source: J Cell Mol Med. 2020 Sep 9;24(19):11198–210. doi: 10.1111/jcmm.15672 (PMC7576294; doi:10.1111/jcmm.15672)
Supplement: Supplementary file 1 — Fig S1‐S2 [file JCMM-24-11198-s001.docx]

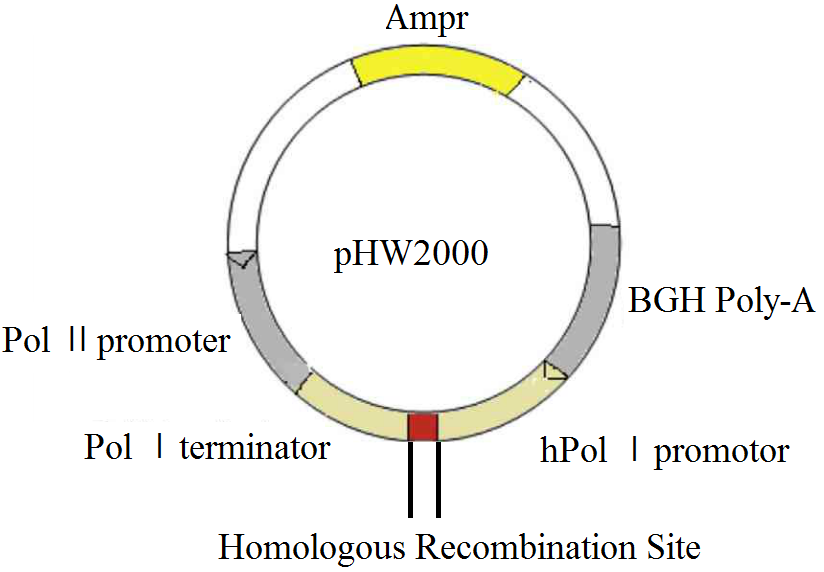


**Figure Supplement 1.** The schematic of plasmid pHW2000 with markings


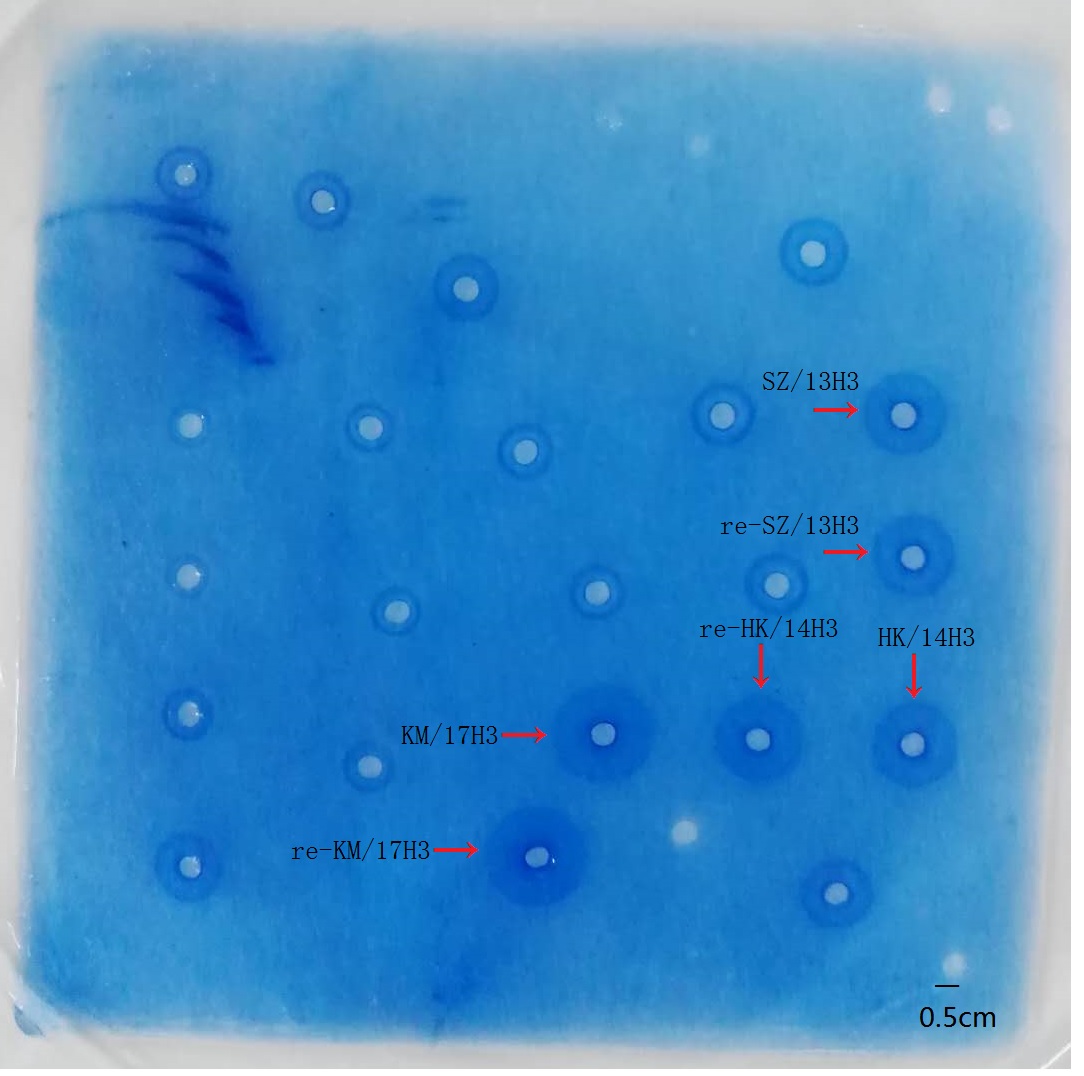


**Figure Supplement 2.** The figure of immunoprecipitated circle of three H3N2 pairs of reassortantment and parental virus strains in the patient sera plate infected with A/Kunming/11/2017 virus.
